# Neighborhood features and depression in Mexican older adults: A longitudinal analysis based on the study on global AGEing and adult health (SAGE), waves 1 and 2 (2009-2014)

**DOI:** 10.1371/journal.pone.0219540

**Published:** 2019-07-10

**Authors:** Julián Alfredo Fernández-Niño, Laura Juliana Bonilla-Tinoco, Betty Soledad Manrique-Espinoza, Aaron Salinas-Rodríguez, René Santos-Luna, Susana Román-Pérez, Evangelina Morales-Carmona, Dustin T. Duncan

**Affiliations:** 1 Public Health Department, Universidad del Norte, Barranquilla, Atlántico, Colombia; 2 Public Health Department, Universidad Industrial de Santander, Bucaramanga, Santander, Colombia; 3 Instituto Nacional de Salud Pública, Cuernavaca, Morelos, México; 4 Spatial Epidemiology Lab, New York University School of Health, New York, New York, United States of America; University of Zurich, SWITZERLAND

## Abstract

A growing body of literature shows that neighborhood characteristics influence older adults’ mental health. Therefore, the aim of this study was to examine the association between structural and social characteristics of the neighborhood, and depression in Mexican older adults. A longitudinal study was conducted based on waves 1 (2009–2010) and 2 (2014) of the Mexican sample from the Study on global AGEing and adult health (SAGE). A street-network buffer around each participant’s household was used to define neighborhood, so that built environment and social characteristics were assessed within it. Depression was ascertained by using an algorithm based on the Composite International Diagnostic Interview. In the analysis, multilevel logistic regression models were constructed separately for each built and social environments measurement, adjusted for socioeconomic, demographic and health-related covariates, and stratified by area of residence (urban versus rural). The results showed that a length of space between 15–45 meters restricted to vehicles was significantly associated with a lower risk of depression in older adults from the urban area (OR: 0.44; IC 95% 0.23–0.83) and the protective association appeared to be larger with increasing space with this restriction, although it lacked significance. Contrarily, the built environment measures were not predictive of depression in the rural setting. On the other hand, none of the variables from the social environment had a significant association, although safety appeared to behave as a risk factor in the overall (OR: 1.48; CI 95% 0.96–2.30; p = 0.08) and rural (OR: 3.44; CI 95% 0.95–12.45; p = 0.06) samples, as it reached marginal significance. Research about neighborhood effects on older adults’ mental health is an emergent field that has shown that depression might be treated not only from the individual-level, but also from the neighborhood-level. Additionally, further research is needed, especially in low- and middle-income countries, to help guide neighborhood policies.

## Introduction

The world population is rapidly ageing mostly due to a higher life expectancy and declining fertility rates [1,2]. This demographic transition raises concerns over several consequences of ageing such as social, economic and health issues. Regarding health, challenges relate to the need of maintaining good health among older adults (people 60 years or older, according to United Nations) and to an increase in the number of older adults affected by mental health problems [2]. As to older adults’ mental health, it is known that depression is the most frequent mental disorder in this age group, and its estimated prevalence can be up to 30% in 65 year old people and older, although it can vary according to sex and setting [3,4]. The importance of depression in the elderly is not solely due to its high prevalence rates, but also to its association with higher morbidity, mortality and disability, and with a higher risk of suicide compared to other age groups [5].

As to the factors associated with depression in older adults, a growing body of literature indicates that the neighborhood environment appears to influence older adults’ mental health [6–11]. The relationship between neighborhood characteristics and older adults’ health (mental and physical) can be explained on the basis that older adults are more vulnerable to the neighborhood’s environment due to multiple reasons [9]. First, not only could older adults be exposed longer to potential harms of the environment, but also the effect of these unhealthy contexts may be more severe in them secondary to elevated biological and psychological vulnerability. Second, neighborhood becomes the most relevant environment for older adults since there is a decrease in the number of other contexts they can move in (work, recreation, etc.); and third, the narrowing social networks of older adults make them rely more intensely on the immediate sources of integration the neighborhood can offer them [9].

In a similar way, the mechanisms by which neighborhood can affect the mental and physical health of older adults are through the modification of health behaviors and levels of psychosocial stress [6]. This effect may come from the two major components of a neighborhood: 1) the material/physical environment (structural dimension) and 2) the psychosocial/social environment (social processes). The first of them refers to the built environment and to the socioeconomic status (SES) of the neighborhood and includes measures of land use type, street connectivity, characteristics of urban design, residential income, education, occupational status, among others [6,10]. It has been documented that older adults living in neighborhoods and localities (which are the smallest administrative division level of the Mexican territory) with a high rate of poverty and deprivation had more depressive symptoms than those who lived in less poor areas or in more affluent environments [8,12]. Besides, not only might the built environment and physical order of the neighborhood promote healthy outdoor physical activities [6,13,14], but socioeconomic advantages of the environment might favor success of its residents [13].

On the other hand, social environment encompasses social capital, social cohesion and collective efficacy [6,15]. Social capital refers to a resource that emerges from the structure of social relationships, “shared norms, and mutual trust that facilitate coordination and cooperation for mutual benefit” [16], and is closely related to social cohesion, which is “the absence of social conflict coupled with the presence of strong social bonds and mutual trust” [17]. These two constructs are protective psychosocial resources against stress and depression by providing affective support and respect among neighbors; by allowing the sharing and transmission of behaviors; and by enabling social and stress control [6,10]. Another related term that might seem very similar to social capital is collective efficacy, which refers to the “linkage of mutual trust and shared expectations for intervening on behalf of the common good”; however, collective efficacy focuses on the shared trust and engagement of the residents of a neighborhood to keep local social control [17].

Conversely to social resources, social disorder can be defined as the “disintegration of processes and structures that maintain order, civility, and safety” [6]. Nevertheless, social disorder is not the only type of disorder, it also exists physical disorder and it should be noted that both of them can result from disadvantage in the neighborhood given by a deficiency of social and economic resources [13]. The lack of order (physical or social) in a neighborhood is reflected by abandoned buildings, filth, graffiti, vandalism, noise, high crime rates, conflicts among residents, public drug use, etc.; and these situations in turn produce a threatening environment suggestive of high risk of danger and disrespect of people and their belongings [13]. Social disorder can lead to depression through several mechanisms such as the continuously increased levels of stress, which trigger a sustained adrenal response; the discouraging of physical activity because of fear of being assaulted; and the resulting limited social contact among residents [13].

This brief review revealed that research about neighborhood effects on older adults’ mental health is scarce [11,18] and that most studies are cross-sectional and have been conducted in high-income countries [19]. This results in evidence with reverse causality limitations and in a low availability of investigations of this type in low- and middle-income countries like Mexico, where the social context and the composition of the neighborhoods are different [20]. In addition, most of available studies do not consider a range of neighborhood-level exposures, which could be important for mental health outcomes among older adults, such as social cohesion, trust and safety.

There are mixed findings for studies examining the association between neighborhood characteristics and older adults’ mental health, which may be explained, at least partly, by the neighborhood definition used, since it’s been demonstrated that this aspect influences the measure of neighborhood exposure [21]. Previously, it has to be noted that neighborhood is a vague and “amorphous” concept that can always be redefined because its construction is based on context, personal experiences and ideals of what a neighborhood should be; therefore, it can vary even among people living in the same space [22,23]. From the point of view of urban sociology, a neighborhood can be defined as a “subsection of a larger community, a collection of both people and institutions occupying a spatially defined area influenced by ecological, cultural, and sometimes political forces” [24]. Another definition regards neighborhoods as “geographical places that can have social and cultural meaning to residents and nonresidents alike and are subdivisions of large places” [25].

From an operational perspective, a neighborhood can be defined through several methods such as using administrative defined area units (census tracts) or egocentric areas like circular buffers, street network buffers or polygon-based buffers [26]. Of the egocentric options, the street network buffer, defined as a “buffer of a specified width placed around a line-based road network”, has several advantages compared to the others [26]. For example, it may assess more accurately the built environment’s factors that influence walking; it is more useful in areas without grid street networks; and is less sensitive to the skew that produces the single use of land (e.g., commercial and industrial) because it does not include inaccessible areas by walking [26]. The latter contrasts with the circular buffer and the polygon method, where a large proportion of these measures may be represented by an area that is not accessible to the pedestrian, but is still used to estimate the built environment measures [21,26].

Additionally, neighborhood effects studies on older adults’ mental health showed that studies about this subject have consistent results regarding some structural characteristics, although there are diverse and even contradictory results about some other features. For example, studies agree on the significant association between disadvantaged neighborhoods, given by greater poverty and less affluence, and depressive symptoms and less social activity in older adults [7,8,11]; but conversely, there are mixed findings in aspects like neighborhood ethnic composition, social environment and perceived environment problems [19]. For all these reasons, the aim of this study was to examine the association between structural and social characteristics of the neighborhood, defined by egocentric buffers, and depression in Mexican older adults.

## Methods

### Study population

The Study on Global Ageing and Adult Health (SAGE) is a multi-country, longitudinal study whose main objective is to generate valid and reliable health data about adult and older adult populations, and to collect information on the ageing process [27,28]. Low- and middle-income countries are: Mexico, China, South Africa, Russian Federation, Ghana and India, all of which were selected because of their wide demographic and economic variability [27,29]. SAGE consists of multiple waves: wave 0, which is the same World Health Survey conducted in 2002–2003 and also served as the baseline for the study; wave 1, collected in 2009–2010; and wave 2 implemented in 2014. In the two latter, the same individual and household questionnaires were applied to participants and also had geocoding/GPS information of the selected households, which is why they were selected for the present research. The design SAGE has been described in detail elsewhere [28].

Regarding the sampling procedure, samples were drawn from a national sampling frame using a stratified, multi-stage cluster design which yielded nationally representative samples of people 50 years and older [28]. In Mexico’s case, wave 1 consisted of 5.448 people and had the lowest response rates (59% and 53% at the household and individual level, respectively) due to the short time for data collection that did not allow revisiting whenever the interviewers did not find the selected respondent [27,28]. However, response rates were higher in wave 2: 83% and 81% at the household and individual levels, respectively; and the main reason for lack of response was the inability to locate the household [30].

A longitudinal study was constructed using waves 1 and 2, so that wave 1 served as baseline and wave 2, as follow-up. The inclusion criteria for selecting the sample were being 55 years or older and having data at follow-up. The criteria about age was set as described in order to include not only people who already were older adults in the baseline, but also individuals who became older adults during the study period. On the other hand, people were excluded from the research if they were classified as having depression in the baseline (wave 1).

### Measures

#### Dependent variable

Depression was assessed in waves 1 and 2 by means of self-report of a medical diagnosis of depression and of the application of an algorithm that is based on the version of the World Survey of Mental Health of the Composite International Diagnostic Interview (CIDI) and has been used in previous research [31]. The algorithm uses SAGE questions about depressive symptoms in the 12 months before the interview to construct two sets: set A and set B. Set A encompasses items about depressive mood, loss of interest and decreased energy, and the duration of these symptoms. Set B is based on other depressive symptoms like loss of appetite, slow down thinking, sleeping problems, concentration difficulties, and death thoughts. According to this algorithm, a person is said to have a possible major depressive episode in the previous 12 months if his/her score is at least 2 and 4 in sets A and B, respectively. Based on the above, an individual was regarded as depressed if reported to have a history of medical diagnosis of depression or if presented a possible depressive episode according to the algorithm. Depression was assessed in wave 1 (the baseline for this study) in order to exclude those people who were classified as depressed, so that remaining participants were depression-free at the beginning of the study and the temporality criteria of causality was accomplished. This enabled the estimation of depression cumulative incidence.

#### Independent variables

Built environment: An egocentric approach was used to define neighborhood. Specifically, a Network Dataset of the road network was constructed from the information of the National Electoral Institute of Mexico, 2015 (Instituto Nacional Electoral) and by using the Network Analyst tool from ArcGIS 10.0. Then, built-environment features were ascertained by using the official data on sidewalk availability in each block (data taken from the Household National Inventory–Inventario Nacional de Viviendas–, 2016). These measurements were the total length (in meters) of street space: 1) intended for pedestrian traffic; 2) with sidewalks; 3) with free access to people; 4) with restricted access to vehicles (as in housing complexes or historical centers); 5) covered with pavement, concrete or paving stone; 6) with street lighting; 7) with trees; and 8) without peddlers. The isochrones method was performed to estimate Manhattan distances (non-linear distances) by starting from the participant’s residence location. Thereby, road network buffers, which spanned 50 meters around the axis of the street network and were up to 950 meters long, were constructed for each subject, as done in previous research [26]. The GIS information was obtained and calculated with Arc-GIS 10.0. It is worth noting that for display purposes, measures of the built environment will be presented as total meters divided by 100, so that 10000 meters will be simplified to 100 meters (per a hundred times).

Social environment: Three variables were constructed based on the questions from the social-cohesion section of the SAGE dataset, as was done in previous research: social capital, trust and solidarity, and safety perception [32]. Social capital was assessed through four items related to the frequency with which the individual had attended a public meeting, met personally with a community leader, attended an organizational meeting and worked with other people from the neighborhood to fix something in the last 12 months. The answers ranged from never to daily and were dichotomized into 1 if the reported frequency was once or twice per month or more, and 0 otherwise. Then, a count of the total positive answers was made, so that the score was categorized into low (0), medium (1) and high (2–4).

Trust was evaluated with the item that asked to what extent participants trusted in people from their neighborhoods; possible answers included "to a very great extent"; “to a great extent”; “neither great, nor small extent”; “to a small extent” and "to a very small extent". People were classified as trusting in their neighbors if reported to trust them to a very great or to a great extent; otherwise, they were said to have no trust in them; this yielded a dichotomous variable (yes vs. no). Finally, safety perception was ascertained with three questions: two of them regarding how safe the person felt from violence and crime when alone at home, and when walking down the street in the dark. The answer options were “completely safe”; “very safe”; “moderately safe”; “slightly safe” and “not safe at all”, so that a participant reporting feeling completely safe or very safe was given a point. The third item was a yes/no question that asked whether the person or someone from their house had been a victim of assault; if the answer was no, another point was given; this way the score ranged from 0–3 and the degree of safety was categorized into low (0), medium (1) and high (2–4).

#### Covariates

Health-related covariates: Multimorbidity was evaluated in two ways. The first of them was by means of self-report of medical diagnosis of some conditions assessed in the individual questionnaire: arthritis, stroke, angina, diabetes, chronic lung disease, asthma, hypertension or cataracts. The second was by applying four additional algorithms to determine if the person had significant symptoms indicative of arthritis, angina, chronic lung disease or asthma in spite of not having a medical diagnosis. These mentioned algorithms have been used previously to estimate the prevalence of the listed chronic conditions [31]. This way, according to the WHO definition, [33] a person was said to have multimorbidity if reported two or more chronic conditions, and these conditions could be ascertained from the self-report or from the algorithms.

Regarding the functional limitations, waves 1 and 2 applied questions based in the WHODAS 2.0, which has been previously validated in low- and middle-income countries [34]. This way, the difficulty for performing several activities in the last 30 days was ascertained. These included sitting for long periods, walking 100 m, standing up from a sitting position, standing for long periods, climbing one flight of stairs, stooping/kneeling/crouching, picking up things with fingers, taking care of household responsibilities, joining community activities, extending arms above the shoulder level, concentrating for 10 min, walking a long distance, bathing, getting dressed, carrying things, moving around inside home, eating, getting up from lying down, getting to and using the toilet and getting to somewhere. These questions have five answer options: none, mild, moderate, severe or extreme/cannot do, so that a person was classified as having functional limitations if reported to have a severe or extreme difficulty for performing any of the listed activities.

Demographic and socioeconomic covariates: Other variables considered for the analysis were sex; age groups (55–59, 60–69, 70–79 and 80+ years); marital status (has a permanent partner, if married or cohabiting vs. no permanent partner, if widowed, divorced, single); educational level (no schooling, complete elementary education, complete high school and complete college studies); area of residence (rural versus urban); and work status (currently working, not currently working and have never worked). The socioeconomic status was determined at the household level through an assets index, which was derived from a dichotomous hierarchical ordered probit model; then, quintiles were obtained [35].

With respect to the social networks, they were assessed by means of a mix of structural and functional features, so that a score was obtained when evaluating four items: marital status, attending religious services, participating in clubs and trusting other people. These items were dichotomized and assigned a point if positive, so the sum of the answers ranged from 0–4, where higher scores represented more social networks [36].

Since the information necessary to construct a deprivation index at the neighborhood level in Mexico is limited, the deprivation index at the municipality level was used as a proxy. This index is a composite measure and it encompasses nine socioeconomic indicators: percentage of individuals aged 15 and above who are illiterate or lack elementary school; percentage of households with any level of overcrowding, soil floors, no water pipeline, no electricity and no drainage; percentage of individuals earning less than two minimum wages; and percentage of individuals dwelling in communities with less than 5,000 inhabitants. This way, four main socioeconomic dimensions are considered: education, household characteristics, work-related income and rurality. The index reflects the degree of marginalization and social exclusion in the different geographic areas, and is a continuous variable in which higher values correspond to higher deprivation levels [37,38].

All covariates were ascertained in wave 1 and were included in the analysis based on previous research [7,8,18,39–41].

### Statistical analysis

In the univariate analysis, quantitative variables were described with central tendency measures (mean and median) and dispersion measures (standard deviation and interquartile range), and with graphic methods. In addition, categorical variables were described with proportions and 95% confidence intervals; the cumulative incidence of depression was estimated in wave 2. In the bivariate analysis, the association of the predictors with the incidence of depression was evaluated with the Mann-Whitney U test for the continuous variables and with the Chi-square and the Fisher’s exact tests for the categorical predictors. Additionally, correlation between built environment variables was estimated using Spearman’s rho, which showed a high correlation between all of them. Considering the potential collinearity due to the theoretical close link between built environment measures, and because the main interest was to identify specific associations, we decided to run separate models for each of the built environment variables.

The multivariable analysis was conducted using multilevel logistic regression models with fixed-effects at the state level and clusters at the municipality level. Fixed effects at the state level were included to account for unobserved characteristics of the 32 Mexican states. Each of the built environment measurements were used as main exposure variables; and age, sex, income index, functional limitations, area of residence and the deprivation index of the municipality were included as adjustment covariates. Furthermore, in addition to constructing models for the whole sample, a stratification by area of residence was performed, since it has been reported that built environment measures might not have the same performance in the rural area as in the urban one [42]. This way, multiple categories were constructed for those variables whose point estimates varied across urban and rural areas, which were used to perform the regression analysis again. The cutoff points to establish these categories were based on the scatter plots of the linear prediction of depression against the selected variables (which were derived from the initial models); so that these thresholds were placed where cutting the cloud of points could yield a different slope of fitted values and still maintain enough observations within each category. For the analysis of the social environment, models were adjusted for all the listed covariates in the Variables section. All the constructed models used state as the second aggregation level. In addition, interactions of the neighborhood characteristics with sex, age (young old vs oldest old) and socioeconomic status were explored by means of stratified analysis. Finally, some complementary analysis were performed, which consisted of a cross-sectional analysis of the subset of the baseline sample who also had a follow-up in wave 2; of the initial models additionally adjusted for depression at baseline, rather than excluding depressed people from wave 1; and a sensitivity analysis comparing individuals with and without complete information to evaluate potential differential losses. All statistical analysis were performed with STATA 15.

### Ethics statement

This research was a secondary analysis of SAGE’s waves 1 and 2, and used data that is publicly available upon formal request to the World Health Organization Data Archive, so that ethical approval from an ethics committee was not necessary. Permission to use data from these waves was obtained from the SAGE-team through the online platform and strict measures were taken to preserve the confidentiality of the GPS information. The original study was approved by the World Health Organization Ethics Committee and all participants provided their written informed consent to be included in the study.

## Results

Applying the inclusion and exclusion criteria yielded a sample of 1524 participants, although 996 (65.35%) constituted the analytical sample because they had complete data. This analytical sample was distributed in 31 groups at the state level, with the lowest and highest number of observations within groups being 4 and 111, respectively. In addition, the average number of observations per group was 35.19, and there were no singletons. Additionally, most of the sample was female (56.83%; CI 95% 53.72–59.88%), was 60–69 years old (46.29% CI 95% 43.20–49.40%), had elementary education (65.96%; CI 95% 62.96–68.85%) and a permanent partner (65.66%; CI 95% 62.65–68.55%). The overall cumulative incidence of depression was 10.64% (CI 95% 8.87–12.72%). As to the health-related variables, 28.92% (CI 95% 26.18–31.82%) and 38.76% (CI 95% 35.77–41.83%) had multimorbidity and functional limitations, respectively. Regarding the measures of the neighborhood, it was found that the highest mean values of length were those of space for pedestrian traffic (mean = 408.00; sd = 218.75); for free access to people (mean = 332.74; sd = 250.86), and without peddlers (mean = 309.99; sd = 233.62). Contrarily, the lowest average length was that of space restricted to vehicles (mean = 15.04; sd = 25.28). With respect to the social environment measurements, most participants reported a low degree of social capital (80.52%; CI 95% 77.94–82.87%) and a high level of safety (58.53%; CI 95% 55.44–61.56%), while only 44.08% (CI 95% 41.01–47.18%) of individuals reported to trust their neighbors. Table 1 shows the complete univariate analysis.

**Table 1:** Sociodemographic, health-related and neighborhood characteristics of the Mexican older adults from SAGE Waves 1 and 2, 2009–2014.

Baseline variables	Total (n = 996) n (%)
Presence of depression ^a^	106 (10.64)
**Neighborhood physical environment**	
Total length in meters of space per 100 meters (mean and sd) ^b^	
*For pedestrian traffic*	408.00 (218.75)
*Sidewalks*	252.23 (234.62)
*Free access to people*	332.74 (250.86)
*Restricted to vehicles*	15.04 (25.28)
*With public lighting*	306.07 (240.07)
*Covered with concrete*	286.40 (242.80)
*With trees*	236.67 (206.49)
*Without peddlers*	309.99 (233.62)
**Neighborhood social environment**	
Social capital (score)	
*Low (0)*	802 (80.52)
*Medium (1)*	119 (11.95)
*High (2–4)*	75 (7.53)
Trust and solidarity	
*No*	557 (55.92)
*Yes*	439 (44.08)
Safety (score)	
*Low (0)*	47 (4.72)
*Medium (1)*	366 (36.75)
*High (2–4)*	583 (58.53)
**Sociodemographic**	
Female sex	566 (56.83)
Age (years)	
*55–59*	181 (18.17)
*60–69*	461 (46.29)
*70–79*	284 (28.51)
*80+*	70 (7.03)
Permanent partner	654 (65.66)
Educational level	
*No formal schooling*	165 (16.57)
*Elementary*	657 (65.96)
*Secondary*	92 (9.24)
*College*	82 (8.20)
Currently working	
*No*	269 (27.01)
*Yes*	303 (30.42)
*Have never worked*	424 (42.57)
Urban area	729 (73.19)
Income quintile	
*1*	198 (19.88)
*2*	204 (20.48)
*3*	180 (18.07)
*4*	221 (22.19)
*5*	193 (19.38)
Social networks	
*0*	72 (7.23)
*1*	331 (33.23)
*2*	425 (42.67)
*3*	150 (15.06)
*4*	18 (1.81)
**Health-related**	
Multimorbidity	288 (28.92)
Functional limitations	386 (38.76)

Results are presented by columns

^a^ This is the only variable measured in wave 2 (incident cases)

^b^ Measurements of the street network buffer. Estimations are presented as total meters divided by a 100 meters

The bivariate analysis showed a significant association between the incidence of depression and sex (p<0.001), work status (p = 0.03), multimorbidity (p<0.01) and functional limitations (p<0.01). There were no significant associations between depression and any of the built and social environment measures of the neighborhood, except for the total length of space with public lighting, which was marginally significant (p = 0.08).

Regarding the multivariable analysis of the cohort (Table 2), it was found that none of the built environment variables were associated with the cumulative incidence of depression in older adults in the global model. However, the point estimates of the space with sidewalks, restricted to vehicles and with trees varied largely when stratifying by area of residence, with the space with vehicle restriction being a marginally significant protector in the urban area (OR: 0.98; CI 95% 0.97–1.00). In addition, it was seen that the built environment measures were not predictive in the rural setting, so it was decided to perform a further analysis of the previously listed variables only in the urban area.

**Table 2:** Association between the neighborhood physical and social environment and the incidence of depression in Mexican older adults from SAGE Waves 1 and 2, 2009–2014.

Baseline variables	Overall (n = 996)		Rural (n = 267)		Urban (n = 729)	
	OR (CI 95%)	p	OR (CI 95%)	p	OR (CI 95%)	p
**Neighborhood physical environment (total length of space per 100 meters)** ^**a**^						
**Model 1**						
For pedestrian traffic	1.00 (0.99–1.00)	0.90	1.00 (0.99–1.00)	0.97	1.00 (0.99–1.00)	0.98
**Model 2**						
Sidewalks	1.00 (0.99–1.00)	0.66	0.87 (1.36e^-9^–5.59e^8^)	0.99	1.00 (0.99–1.00)	0.36
**Model 3**						
Free access to people	1.00 (0.99–1.00)	0.97	0.96 (<0.01–3978.93)	0.99	1.00 (0.99–1.00)	0.51
**Model 4**						
Restricted to vehicles	0.99 (0.98–1.00)	0.09	0.005 (—)	0.99	**0.98 (0.97–1.00)**	**0.05**
**Model 5**						
With public lighting	1.00 (0.99–1.00)	0.60	0.95 (9.23e^-7^–968870.40)	0.99	1.00 (0.99–1.00)	0.22
**Model 6**						
Covered with concrete	1.00 (0.99–1.00)	0.62	0.91 (6.16e^-10^–1.36e^9^)	0.99	1.00 (0.99–1.00)	0.37
**Model 7**						
With trees	1.00 (0.99–1.00)	0.47	0.56 (4.57e^-64^–6.88e^62^)	0.99	1.00 (0.99–1.00)	0.17
**Model 8**						
Without peddlers	1.00 (0.99–1.00)	0.93	0.96 (<0.001–4616.60)	0.99	1.00 (0.99–1.00)	0.44
**Neighborhood social environment** ^**b**^						
**Model 9**						
Social capital (score)						
*Low (0)*	Ref.		Ref.		Ref.	
*Medium (1)*	0.97 (0.50–1.89)	0.92	0.70 (0.10–4.89)	0.72	0.61 (0.25–1.47)	0.27
*High (2–4)*	0.94 (0.39–2.28)	0.90	1.53 (0.19–12.58)	0.69	0.78 (0.29–2.14)	0.63
**Model 10**						
Trust and solidarity						
*No*	Ref.		Ref.		Ref.	
*Yes*	0.84 (0.56–1.27)	0.41	0.46 (0.14–1.55)	0.21	0.88 (0.55–1.43)	0.62
**Model 11**						
Safety (score)						
*High (2–4)*	Ref.		Ref.		Ref.	
*Medium (1)*	1.48 (0.96–2.30)	0.08	3.44 (0.95–12.45)	0.06	1.30 (0.75–2.23)	0.35
*Low (0)*	1.69 (0.63–4.46)	0.30	2.93 (0.22–39.17)	0.42	1.40 (0.52–3.77)	0.50

^a^ Models with state as the second aggregation level and adjusted for sex, age, income index, functional limitations and margination index of the municipality. Overall models also adjusted for area of residence.

(—) Non-estimable

^b^ Models with state as the second aggregation level and adjusted for age group, sex, marital status, education level, income quintile, work status, area of residence, social networks, multimorbidity, functional limitations and margination index of the municipality.

To achieve this, several steps were followed: 1) the linear prediction of the incidence of depression (logit) was predicted for the models using the space with sidewalks, with trees and restricted to vehicles as main predictors. 2) Those linear predictions were plotted against their correspondent predictor in its continuous form (S1–S3 Figs). 3) The cutoff points to create the categories for the built-environment variables were chosen based on the distribution of the scatter points along the x-axis; this way a regression model was performed in each category, yielding very different slopes from the ones obtained in the general model (S4–S6 Figs). Additionally, different cut-off points were tested, but the ones that allowed a sufficient sample within each category, had the lowest log-likelihood and made sense for practical use were the ones that were kept for display in Table 3. Anyhow, the other tested cutoff points yielded similar results and pointed towards the same conclusions (S1 Table of the Supporting Information file). The scatter plots used to select the cutoff points to create the categories for each variable and the sensitivity analysis of these are shown in the Supporting Information file.

**Table 3:** Association between three physical environment measurements and the incidence of depression in Mexican older adults from the urban area from SAGE Waves 1 and 2, 2009–2014.

Urban area (n = 729)		
Neighborhood measurement	OR (CI 95%)	p
Space with sidewalks (per 100 meters)		
*0 to less than 50*	Ref.	
*50 to less than 250*	0.54 (0.20–1.43)	0.22
*250 to less than 450*	0.55 (0.20–1.53)	0.25
*450 or more*	1.10 (0.42–2.88)	0.85
Space with tress (per 100 meters)		
*0 to less than 100*	Ref.	
*100 to less than 300*	0.42 (0.15–1.23)	0.11
*300 to less than 500*	0.57 (0.19–1.73)	0.32
*500 or more*	0.90 (0.29–2.83)	0.86
Space restricted to vehicles (per 100 meters)		
*0 to less than 15*	Ref.	
*15 to less than 45*	0.45 (0.24–0.85)	**0.02**
*45 to less than 80*	0.51 (0.17–1.55)	0.24
*80 or more*	0.30 (0.06–1.54)	0.15

Models with state as the second aggregation level and adjusted for sex, age, income index, functional limitations and deprivation index of the municipality.

Cutoff points based on the scatter plots

When conducting the models with the categories of the aforementioned variables, it was evidenced that the space with vehicle restriction showed a protective association with the incidence of depression (Table 3). Even more, the model suggested a possible dose-response relationship, so that the more space restricted to vehicles, the more protection it displayed, although only one category had statistical significance (OR_1_: 0.45, CI 95% 0.24–0.85; OR_2_: 0.51, CI 95% 0.17–1.55; OR_3_: 0.30, CI 95% 0.06–1.54). The space with trees also indicated a protective effect, though it was not significant, and the space with sidewalks did not show a clear direction of the association, nor it was significant.

As to the measurements of the social environment of the neighborhood (Table 2), none of these variables were statistically associated with the incidence of depression in older adults, although the analysis suggested that higher levels of social capital had a possible protective association in the urban area (OR_medium_: 0.61, CI 95% 0.25–1.47; OR_high_: 0.78, CI 95% 0.29–2.14). Similarly, trusting in neighbors appeared to be protective in the overall and stratified-by-area models. On the other hand, lower levels of safety showed higher risk of depression in the overall sample and in the rural and urban settings, with the medium level of safety being marginally significant in the overall (OR: 1.48; CI 95% 0.96–2.30) and in the rural (OR: 3.44; CI 95% 0.95–12.45) models. The estimations in the latter were imprecise due to the scarce sample in the rural setting.

Finally, the analysis to explore potential interactions was carried out, although it is worth mentioning that performing stratified analysis to explore interactions reduces the available sample for modeling estimations, which results in imprecise estimates. All the results from this last step of the analysis can be seen in the Supporting Information file.

The sex-stratified analysis evidenced that the space restricted to vehicles seemed to reduce the likelihood of depression only in women, although it was marginally significant and the magnitude of the association was quite small and very close to the null (OR: 0.99; CI 95% 0.97–1.00). In addition, the point estimates suggested that higher levels of social capital and trust in neighbors were protective against depression only in women; while lower levels of safety increased the chances of depression in both men and women. However, neither of these variables reached statistical significance in any case (S2 Table of the Supporting Information file).

On the other hand, for performing the age-stratified analysis, the young old and oldest old categories were created based on the United Nations definition, which classifies individuals 80 years old and older as “oldest old” [2]. Based on the above, the analysis revealed that the space restricted to vehicles seemed to reduce the likelihood of depression in the oldest old group (OR: 0.88; CI 95% 0.77–1.01), although it should be noted that the estimates from the oldest old group were very imprecise due to its very small sample (n = 70). Contrarily, none of the evaluated variables showed a significant association with depression in the young old group (S3 Table).

Lastly, in the analysis stratified by socioeconomic status, categories were created by grouping quintiles 1 and 2 into the “low” category; quintile 3 remained as “medium”; and quintiles 4 and 5 were grouped to form the “high” category. This way, it was found that space with free access to people (OR: 1.00; CI 95% 1.00–1.01), with public lighting (OR: 1.00; CI 95% 1.00–1.01) and covered in concrete (OR: 1.00; CI 95% 0.99–1.00) were statistically and marginally significant respectively. However, the point estimates and the CI 95% were the null value or very close to it. On the other hand, a high level of social capital significantly decreased the likelihood of depression in individuals from a low socioeconomic status (OR: 0.08; CI 95% 0.01–0.53); and lower levels of safety increased the odds of depression in older adults from a high socioeconomic status, with the medium level being statistically significant compared to a high level of safety (OR: 2.90; CI 95% 1.09–7.71). Results shown in S4 Table.

### Complementary analysis

Complementary analysis showed similar results to those displayed in Table 2 (S5 and S6 Tables). In addition, some sensitivity analysis were performed to assess for missing data and the results of the regression models when incorporating the complex sampling design. In the missing data case, the sensitivity analysis was made to ascertain differential losses of data variables under study given that the analytical sample was roughly two thirds of the original sample (S7 Table). This way, the affected variables were adjusted for in the regression models. In the second sensitivity analysis, multiple logistic regression models were run incorporating the complex sampling design of the original study (SAGE), so that the strata and sampling weights were specified (S8 Table). The results of these analyses are presented in the Supporting information file.

## Discussion

With the aim of examining the association between physical and social characteristics of the neighborhood and depression in Mexican older adults, the present research found that vehicular restriction was a protective factor against the incidence of depression in older adults from urban areas. Even more, it seemed to be a possible dose-response relationship, so that larger lengths of space restricted to vehicles showed a higher protection. These results are consistent with previous research that have found significant associations between mental well-being and traffic safety [40,43] and unsafe traffic and lack of help within neighbors [40]. Furthermore, a qualitative study showed that older adults identified traffic safety as a very important factor that affected their health [44]. Contrarily, the present findings contrast with other investigation where green areas were associated with depressive symptoms [45].

There are multiple potential mechanisms for the found association and they come from the benefits of pedestrianization, which refers to impede the access of motor vehicles to a determined area [46]. For example, the space restricted to vehicles promotes physical activity (reflected in the increasing number of journeys on foot), which in turn has been shown to alleviate depressive symptoms [47–49]. Additionally, the pedestrianization of streets stimulates the practice of other activities since different land-use other than for traffic can be implemented, so it becomes an opportunity to personally interact and communicate with other people, thereby increasing social interaction [46,47,50,51]. It has to be reminded that social networks and social contact are important factors that influence the onset of depression [52,53]. As to the personal safety perception, restriction of vehicles improves it because it reduces the rates of crime and of traffic accidents involving pedestrians, so the elderly can comfortably and safely walk, which derives in the reduction of stress levels while walking [46,47]. Finally, other pathways by which the vehicle restriction might improve mental health (and depression) is through the reduction of air pollution and noise, since these factors are associated with cardiovascular diseases, which at the same time are highly associated with depression [47,51,52,54].

In addition to the previously described, exploration of interactions showed that women and “oldest-old” individuals seemed to benefit more from the space restricted to vehicles compared to men and young olds, respectively; although the reduction of the likelihood of depression was more evident in the age-stratified analysis. Explanations for these findings can also come from the benefits of pedestrianization, specifically, from the increase of social interaction and of safety. Firstly, it has been described that gender differences in contextual effects might be due to 1) different perceptions of their environment; 2) different exposure to certain aspects of the neighborhood; and 3) differential vulnerability to those aspects [55]. For example, women are reported to have a higher social interaction and more social networks within their neighborhoods [56], therefore, they might experience a greater benefit from a neighborhood characteristic that promotes social interaction and communication, which are protective factors against depression. This would be in accordance with previous research that have found that neighborhood social features, such as social cohesion and social capital mediated the association between neighborhood disadvantage/physical environment and depression [57,58]. On the other hand, ageing comes with a decline in health and changes in functioning, such as diminished cognitive capacity and senses, which accentuates at oldest ages, so that the elderly (especially the oldest old) are more prone to traffic accidents [59,60]. This way, the enhanced safety given by the space with vehicle restriction might alleviate the described situation, since it reduces the rate of traffic accidents where pedestrians are involved, which allows older adults to walk without fearing a traffic accident.

Moreover, the analysis also suggested a possible association between the incidence of depression and some features of the social environment in both urban and rural areas, although the estimations did not have statistical significance, possibly due to a problem of lack of power. In particular, individuals who trusted their neighbors seemed to have a lower risk of depression, while people who perceived higher levels of crime in their neighborhoods appeared to have a higher risk of depression compared to those who consider it safe. The lack of significant associations contrasts with other investigations where crime, unwillingness of neighbors to help each other, neighborhood safety and social cohesion were significantly associated with higher and lower depressive symptoms in older adults, respectively [40,41,61,62].

However, when stratifying by socioeconomic status, a high level of social capital was a significant protector against depression in low socioeconomic status older adults, while a medium level of safety significantly increased the likelihood of depression in high socioeconomic status individuals. These findings might relate to the adequacy of social and economic resources of people from different socioeconomic statuses, so that different aspects of social environment could be more or less beneficial in certain settings. However, this should be further investigated to extend the knowledge on this aspect.

Considering all of the above, it appears that the social environment of the neighborhood could have a larger influence on mental health outcomes, such as depression. This could be hypothesized since not only the analysis suggested that social capital and safety were associated with the likelihood of depression in Mexican older adults from low and high socioeconomic status, respectively; but also it showed that a built environment characteristic that promotes social interaction reduced the likelihood of depression in older adults from urban areas. Additionally, it has been described that aspects from the social environment, such as social capital and social cohesion mediate the influence of structural characteristics of the neighborhood on the health of its residents [63]. This way, it could be suggested that a neighborhood that enhances social ties and networks, and provides social resources that help buffer stress levels might have a better impact on mental health. Moreover, the decay of the built environment (which has been associated with depressive symptoms in previous research) can reflect a problematic social environment that lacks social control and where social disorder prevails [10,13]. On the contrary, physical activity and other outcomes related to the fulfillment of it, i.e. obesity, hypertension, diabetes, might be more influenced by the “walkability” of the neighborhood, in which the built environment plays a major role [10,64].

Since conducting randomized trials for studying neighborhood effects is largely difficult, natural experiments become a very useful tool, in spite of the intervention not being randomly assigned [65,66]. Therefore, for future investigations, researchers should take advantage of the natural experiments occurring within neighborhoods, especially of those related to the transformation or intervention of the built environment [67]. The above in order to evaluate the impact on health indicators, including the ones related to mental health; in the specific case of Mexico, research should focus on the impact of the infrastructure modification in the health of its inhabitants.

This study has important strengths and limitations. With respect to the strengths of this research, they relate to the inclusion of both built and social environment features, so that built environment and social context were considered in the analysis; also, the measurements of the built environment were done objectively through the usage of GIS information. Furthermore, the individual information of participants was obtained through the application of previously validated and calibrated instruments, which allowed adjusting for the main individual-level confounders in the multivariable analysis. Additionally, since several hypothesis tests were conducted in the multivariable analysis, the obtained results were reviewed and re-analyzed controlling for the False Discovery Rate and our conclusions remained the same. Notwithstanding, we consider that further investigation should be done to confirm the findings from this study and continue this line of research. On the other hand, this is a longitudinal study, so that the 5-year incidence of major depression was modelled in the analysis. Lastly, this research not only adds up to the few available researches about neighborhood effects on human health in Latin America with a nationally representative sample [68], but is also the first investigation to our knowledge about neighborhood effects and mental health with a nationally representative sample in Mexico, since the other studies about neighborhood effects in Mexico are focused on different outcomes [69,70]. Moreover, the study of neighborhood effects on mental health is an emergent field as most of studies are conducted on physical activity, obesity or chronic disease. In addition, the present investigation contributes filling the gap regarding longitudinal studies on neighborhood effects in a Latin sample of older adults, as has been described in previous research [61].

Regarding the limitations of this study, the main concern arises from the fact that the used data had very few observations within each neighborhood, that is, there were scarce participants coming from the same neighborhood. This situation is due to the design of the primary study (SAGE) because its sample was intended to be nationally representative, which does not guarantee representativity at lower levels such as the town-level and the neighborhood-level. The described lack of sufficient individuals derives in a larger within-variance than the between-variance, which hinders the correct distinction between contextual and compositional effects of the neighborhood on the mental health of older adults. Additionally, the lack of sufficient individuals within the same neighborhood made us use the state as the second aggregation level, making it the Primary Sampling Unit (PSU) for this specific research. This, in turn, differs from the PSU established for SAGE, which prevented us from being able to account for the complex design of the original research simultaneously to the multilevel analysis. However, a sensitivity analysis was performed by running multiple logistic regression models after having specified the complex sampling design of SAGE. While few of the results were sensitive to the choice of incorporating sampling design, our overall conclusions remained unchanged. Besides, it can be considered that the most important aspect about this investigation was to account for the correlation structure within clusters of the second aggregation level (the states), not for the PSU of the original study, which was accomplished by means of the multilevel analysis. Furthermore, there was a reduction of 34.65% in the sample due to missing data. This limitation could be a result of the low response rate from wave 1, which was caused by a short time for fieldwork, so there were not many opportunities to revisit participants in case they were not found during the first contact [28]. Based on the above, it is reasonable to think that the losses were random. However, a sensitivity analysis was performed to compare individuals with and without complete data, so that those variables that were found to be associated with the completeness of information were adjusted for in the multivariable analysis. Additionally, neighborhood was defined through a street-network buffer, which is less sensitive to destinations people actually go to and to the real environmental factors older adults are exposed to in their neighborhoods, as demonstrated by previous studies performed with a neighborhood definition based on GPS data, since it is a presumed residential area where individuals mobilize [14,71]. Notwithstanding, the street-network buffer has several advantages over the other residential buffers [26]. Furthermore, a problem of lack power to detect associations might be present since the incidence of the depression cases was not as high as expected and because the built environment measurements lacked variability and tended to be skewed to the left (towards the zero), especially in the rural settings. This last data difficulty, along with the fact that built-environment measures from the urban area are not entirely appropriate for studying neighborhood effects in the rural area [42,72,73], hindered the correct analysis in the rural setting. Therefore, the obtained results relating to the physical context in the urban area might not be applicable to rural areas. It should also be noted that depression was partly measured with an algorithm based on depressive symptoms, so that there might be misclassification of the outcome since it was not possible to perform a clinical assessment of participants. Moreover, confounding might be present in the estimations since it was not possible to include data on some variables that the literature underscores as important when performing the research, such as the type of land use that predominates, the number of years living in the neighborhood, and the proportion of older adults living in the neighborhood of the participants. In addition, a direct measure of the socioeconomic status of the neighborhood, like the deprivation index specific to the neighborhood, could not be used in the analysis given the limited availability of this information. Nonetheless, adjustment was made with the deprivation index of the municipality, which is the second administrative level in Mexico and had a correlation of 0.36 (p = <0.001) with a sample of the deprivation index of the neighborhood. Finally, the analysis could not incorporate an objective measure of safety or crime of the neighborhoods because that information was not available to use.

## Conclusions

Investigating neighborhood effects on older adults’ mental health is an emergent field as most of studies are conducted on physical activity, obesity or chronic diseases; however, more research on this aspect is needed since not only world population is rapidly ageing, but also depression is one of the largest contributors of burden disease in this age group. In addition, the study of depression tends to be done at the individual-level, neglecting that the environment where a person develops also plays a key role in the risk of having depression. Therefore, it might be beneficial to not just individually treat each depressed patient, but also make interventions at the neighborhood-level that improve or complement the treatment of this mental disease and prevents its onset. The present investigation contributes to reinforce the knowledge about the association between the built-environment (specifically, traffic safety) and depression in older adults and to fill the gap regarding longitudinal studies on neighborhood effects in a Latin sample of older adults, as has been described in previously (38). Nonetheless, further research on this subject is needed, especially in low- and middle-income countries, to keep evaluating neighborhood characteristics that might be related to older adults’ mental health in order to help guiding policies related to urban planning and neighborhood interventions.

## Supporting information

S1 Fig Scatter plot of the linear prediction of depression in the follow-up (wave2) against the space with sidewalks. (DOCX)

S2 Fig Scatter plot of the linear prediction of depression in the follow-up (wave2) against the space with trees. (DOCX)

S3 Fig Scatter plot of the linear prediction of depression in the follow-up (wave2) against the space restricted to vehicles. (DOCX)

S4 Fig Scatter plot of the linear prediction of depression in the follow-up (wave2) within each category of the space with sidewalks. (DOCX)

S5 Fig Scatter plot of the linear prediction of depression in the follow-up (wave2) within each category of the space with trees. (DOCX)

S6 Fig Scatter plot of the linear prediction of depression in the follow-up (wave2) within each category of the space restricted to vehicles. (DOCX)

S1 Table Sensitivity analysis of other cut-off points for the three physical environment measurements variables. (DOCX)

S2 Table Sex-stratified analysis (using area of residence as an adjustment covariate). (DOCX)

S3 Table Age-stratified analysis (oldest old vs. young old). (DOCX)

S4 Table SES-stratified analysis (quintiles 1 and 2: Low; quintile 3: Medium; and quintiles 4 and 5 high). (DOCX)

S5 Table Cross-sectional analysis of the subsample from wave 1 (baseline). (DOCX)

S6 Table Analysis of the cohort adjusting for depression at baseline. (DOCX)

S7 Table Sensitivity analysis of individuals with and without complete information. (DOCX)

S8 Table Sensitivity analysis of logistic regression models accounting for the complex sampling design of SAGE. (DOCX)
