# Supplementary material for: Neighborhood features and depression in Mexican older adults: A longitudinal analysis based on the study on global AGEing and adult health (SAGE), waves 1 and 2 (2009-2014)
Source: PLoS One. 2019 Jul 10;14(7):e0219540. doi: 10.1371/journal.pone.0219540 (PMC6619793; doi:10.1371/journal.pone.0219540)
Supplement: S5 Fig — (DOCX) [file pone.0219540.s005.docx]

**S5 Figure. Scatter plot of the linear prediction of depression in the follow-up (wave2) within each category of the space with trees**

**
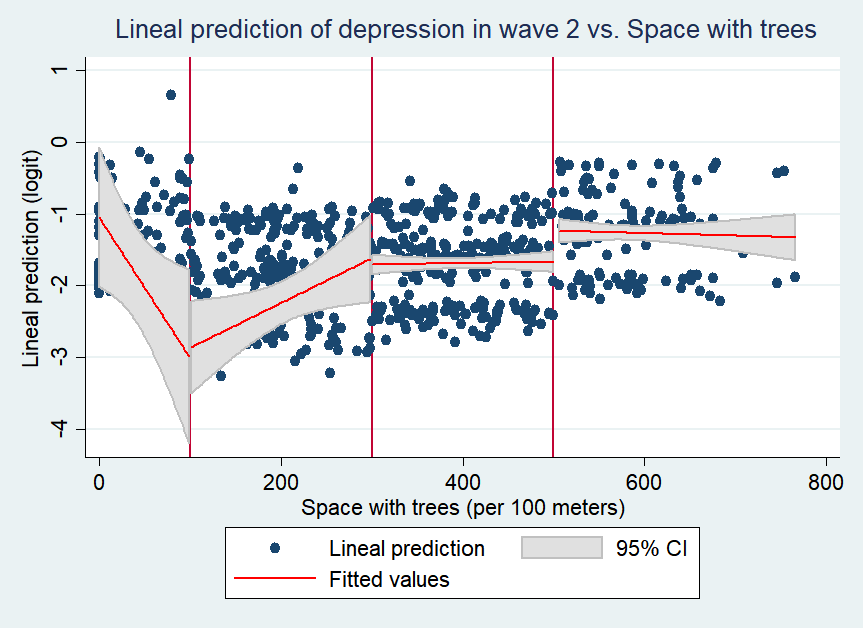
**
