# Supplementary material for: Neighborhood features and depression in Mexican older adults: A longitudinal analysis based on the study on global AGEing and adult health (SAGE), waves 1 and 2 (2009-2014)
Source: PLoS One. 2019 Jul 10;14(7):e0219540. doi: 10.1371/journal.pone.0219540 (PMC6619793; doi:10.1371/journal.pone.0219540)
Supplement: S1 Table — (DOCX) [file pone.0219540.s007.docx]

**S1 Table. Sensitivity analysis of other cut-off points for the three physical environment measurements variables**

| **Urban area (n=729)** | | |
| --- | --- | --- |
| **Neighborhood measurement** | **OR (CI 95%)** | **p** |
| **Space with sidewalks (per 100 meters)** |  |  |
| Set 1 |  |  |
| *0 to less than 100* | Ref. | |
| *100 to less than 300* | 0.64 (0.27-1.52) | 0.31 |
| *300 to less than 500* | 0.55 (0.22-1.36) | 0.19 |
| *500 or more* | 1.39 (0.58-3.35) | 0.46 |
| Set 2 |  |  |
| *0 to less than 70* | Ref. | |
| *70 to less than 200* | 0.62 (0.22-1.75) | 0.37 |
| *200 to less than 500* | 0.49 (0.20-1.22) | 0.12 |
| *500 or more* | 1.22 (0.48-3.11) | 0.67 |
| **Space with tress (per 100 meters)** |  |  |
| Set 1 |  |  |
| *0 to less than 50* | Ref. | |
| *50 to less than 250* | 0.25 (0.06-1.05) | 0.06 |
| *250 to less than 450* | 0.72 (0.18-2.93) | 0.65 |
| *450 or more* | 0.64 (0.16-2.56) | 0.53 |
| Set 2 |  |  |
| *0 to less than 70* | Ref. | |
| *70 to less than 200* | 0.32 (0.08-1.35) | 0.12 |
| *200 to less than 450* | 1.11 (0.30-4.16) | 0.87 |
| *450 or more* | 1.08 (0.29-4.07) | 0.91 |
| **Space restricted to vehicles (per 100 meters)** |  |  |
| Set 1 |  |  |
| *0 to less than 20* | Ref. | |
| *20 to less than 50* | 0.44 (0.21-0.91) | **0.03** |
| *50 to less than 100* | 0.67 (0.23-1.89) | 0.44 |
| *100 or more* | 0.36 (0.04-3.12) | 0.35 |
| Set 2 |  |  |
| *0 to less than 25* | Ref. | |
| *25 to less than 50* | 0.63 (0.29-1.41) | 0.26 |
| *50 to less than 100* | 0.73 (0.26-2.09) | 0.56 |
| *100 or more* | 0.40 (0.05-3.48) | 0.41 |

Models with state as the second aggregation level and adjusted for sex, age, income index, functional limitations and deprivation index of the municipality.

Cutoff points based on the scatter plots
