# Supplementary material for: Neighborhood features and depression in Mexican older adults: A longitudinal analysis based on the study on global AGEing and adult health (SAGE), waves 1 and 2 (2009-2014)
Source: PLoS One. 2019 Jul 10;14(7):e0219540. doi: 10.1371/journal.pone.0219540 (PMC6619793; doi:10.1371/journal.pone.0219540)
Supplement: S2 Table — (DOCX) [file pone.0219540.s008.docx]

**S2 Table. Sex-stratified analysis (using area of residence as an adjustment covariate)**

| **Baseline variables** | **Overall (n= 996)** | | **Male (n= 430)** | | **Female (n= 566)** | |
| --- | --- | --- | --- | --- | --- | --- |
|  | **OR (CI 95%)** | **p** | **OR (CI 95%)** | **p** | **OR (CI 95%)** | **p** |
| **Neighborhood physical environment (total length of space per 100 meters) ^a^** |  |  |  |  |  |  |
| **Model 1** | | | | | | |
| For pedestrian traffic | 1.00 (0.99-1.00) | 0.90 | 1.00 (0.99-1.00) | 0.35 | 1.00 (0.99-1.00) | 0.51 |
| **Model 2** | | | | | | |
| Sidewalks | 1.00 (0.99-1.00) | 0.66 | 1.00 (0.99-1.00) | 0.13 | 1.00 (0.99-1.00) | 0.62 |
| **Model 3** | | | | | | |
| Free access to people | 1.00 (0.99-1.00) | 0.97 | 1.00 (0.99-1.00) | 0.34 | 1.00 (0.99-1.00) | 0.61 |
| **Model 4** | | | | | | |
| Restricted to vehicles | 0.99 (0.98-1.00) | 0.09 | 1.00 (0.98-1.02) | 0.81 | **0.99 (0.97-1.00)** | **0.07** |
| **Model 5** | | | | | | |
| With public lighting | 1.00 (0.99-1.00) | 0.60 | 1.00 (0.99-1.01) | 0.19 | 1.00 (0.99-1.00) | 0.86 |
| **Model 6** | | | | | | |
| Covered with concrete | 1.00 (0.99-1.00) | 0.62 | 1.00 (0.99-1.01) | 0.11 | 1.00 (0.99-1.00) | 0.67 |
| **Model 7** | | | | | | |
| With trees | 1.00 (0.99-1.00) | 0.47 | 1.00 (0.99-1.00) | 0.23 | 1.00 (0.99-1.00) | 0.85 |
| **Model 8** | | | | | | |
| Without peddlers | 1.00 (0.99-1.00) | 0.93 | 1.00 (0.99-1.00) | 0.35 | 1.00 (0.99-1.00) | 0.60 |
| **Neighborhood social environment ^b^** |  |  |  |  |  |  |
| **Model 9** | | | | | | |
| Social capital (score) |  |  |  |  |  |  |
| *Low (0)* | Ref. | | Ref. | | Ref. | |
| *Medium (1)* | 0.97 (0.50-1.89) | 0.92 | 1.59 (0.37-6.92) | 0.53 | 0.81 (0.41-1.61) | 0.55 |
| *High (2-4)* | 0.94 (0.39-2.28) | 0.90 | 1.41 (0.15-13.15) | 0.76 | 0.88 (0.32-2.44) | 0.81 |
| **Model 10** | | | | | | |
| Trust and solidarity |  |  |  |  |  |  |
| *No* | Ref. | | Ref. | | Ref. | |
| *Yes* | 0.84 (0.56-1.27) | 0.41 | 1.38 (0.41-4.61) | 0.60 | 0.76 (0.47-1.24) | 0.27 |
| **Model 11** | | | | | | |
| Safety (score) |  |  |  |  |  |  |
| *High (2-4)* | Ref. | | Ref. | | Ref. | |
| *Medium (1)* | 1.48 (0.96-2.30) | 0.08 | 1.36 (0.36-5.15) | 0.65 | 1.47 (0.83-2.60) | 0.19 |
| *Low (0)* | 1.69 (0.63-4.46) | 0.30 | 3.25 (0.17-61.06) | 0.43 | 1.34 (0.58-3.07) | 0.50 |

^a^ Models with state as the second aggregation level and adjusted for age, income index, area of residence, functional limitations and margination index of the municipality.

^b^ Models with state as the second aggregation level and adjusted for age group, marital status, education level, income quintile, work status, area of residence, social networks, multimorbidity, functional limitations and margination index of the municipality.
