# Supplementary material for: Neighborhood features and depression in Mexican older adults: A longitudinal analysis based on the study on global AGEing and adult health (SAGE), waves 1 and 2 (2009-2014)
Source: PLoS One. 2019 Jul 10;14(7):e0219540. doi: 10.1371/journal.pone.0219540 (PMC6619793; doi:10.1371/journal.pone.0219540)
Supplement: S4 Table — (DOCX) [file pone.0219540.s010.docx]

**S4 Table. SES-stratified analysis (quintiles 1 and 2: low; quintile 3: medium; and quintiles 4 and 5 high)**

| **Baseline variables** | **High (n= 414)** | | **Medium (n= 180)** | | **Low (n= 402)** | |
| --- | --- | --- | --- | --- | --- | --- |
|  | **OR (CI 95%)** | **p** | **OR (CI 95%)** | **p** | **OR (CI 95%)** | **p** |
| **Neighborhood physical environment (total length of space per 100 meters) ^a^** |  |  |  |  |  |  |
| **Model 1** | | | | | | |
| For pedestrian traffic | 1.00 (0.99-1.00) | 0.23 | 1.00 (0.99-1.01) | 0.56 | 1.00 (0.99-1.00) | 0.32 |
| **Model 2** | | | | | | |
| Sidewalks | 1.00 (0.99-1.00) | 0.07 | 1.00 (0.99-1.00) | 0.58 | 1.00 (0.99-1.00) | 0.43 |
| **Model 3** | | | | | | |
| Free access to people | 1.00 (1.00-1.01) | **0.04** | 1.00 (0.99-1.00) | 0.68 | 1.00 (0.99-1.00) | 0.25 |
| **Model 4** | | | | | | |
| Restricted to vehicles | 0.98 (0.96-1.01) | 0.16 | 1.00 (0.95-1.03) | 0.67 | 1.00 (0.98-1.01) | 0.51 |
| **Model 5** | | | | | | |
| With public lighting | 1.00 (1.00-1.01) | **0.03** | 1.00 (0.99-1.01) | 0.54 | 1.00 (0.99-1.00) | 0.53 |
| **Model 6** | | | | | | |
| Covered with concrete | 1.00 (0.99-1.00) | 0.06 | 1.00 (0.99-1.01) | 0.37 | 1.00 (0.99-1.00) | 0.42 |
| **Model 7** | | | | | | |
| With trees | 1.00 (0.99-1.01) | 0.15 | 1.00 (0.99-1.01) | 0.51 | 1.00 (0.99-1.00) | 0.66 |
| **Model 8** | | | | | | |
| Without peddlers | 1.00 (0.99-1.01) | 0.06 | 1.00 (0.99-1.00) | 0.88 | 1.00 (0.99-1.00) | 0.30 |
| **Neighborhood social environment ^b^** |  |  |  |  |  |  |
| **Model 9** | | | | | | |
| Social capital (score) |  |  |  |  |  |  |
| *Low (0)* | Ref. | | Ref. | | Ref. | |
| *Medium (1)* | 1.05 (0.17-6.38) | 0.95 | -- | -- | 0.63 (0.22-1.76) | 0.37 |
| *High (2-4)* | 7.37 (0.92-58.99) | 0.06 | -- | -- | 0.08 (0.01-0.53) | **<0.01** |
| **Model 10** | | | | | | |
| Trust and solidarity |  |  |  |  |  |  |
| *No* | Ref. | | Ref. | | Ref. | |
| *Yes* | 0.50 (0.17-1.42) | 0.19 | 0.34 (0.02-7.55) | 0.50 | 1.27 (0.53-3.02) | 0.59 |
| **Model 11** | | | | | | |
| Safety (score) |  |  |  |  |  |  |
| *High (2-4)* | Ref. | | Ref. | | Ref. | |
| *Medium (1)* | 2.90 (1.09-7.71) | **0.03** | -- | -- | 0.69 (0.25-1.94) | 0.48 |
| *Low (0)* | 1.62 (0.11-23.58) | 0.72 | -- | -- | 1.76 (0.44-7.08) | 0.42 |

^a^ Models with state as the second aggregation level and adjusted for sex, age, area of residence, functional limitations and margination index of the municipality.

(--) Non-estimable

^b^ Models with state as the second aggregation level and adjusted for sex, marital status, education level, age group, work status, area of residence, social networks, multimorbidity, functional limitations and margination index of the municipality.
