# Supplementary material for: Neighborhood features and depression in Mexican older adults: A longitudinal analysis based on the study on global AGEing and adult health (SAGE), waves 1 and 2 (2009-2014)
Source: PLoS One. 2019 Jul 10;14(7):e0219540. doi: 10.1371/journal.pone.0219540 (PMC6619793; doi:10.1371/journal.pone.0219540)
Supplement: S6 Table — (DOCX) [file pone.0219540.s012.docx]

**S6 Table. Analysis of the cohort adjusting for depression at baseline**

| **Baseline variables** | **Overall (n= 1180)** | | **Rural (n= 310)** | | **Urban (n= 870)** | |
| --- | --- | --- | --- | --- | --- | --- |
|  | **OR (CI 95%)** | **p** | **OR (CI 95%)** | **p** | **OR (CI 95%)** | **p** |
| **Neighborhood physical environment (total length of space per 100 meters) ^a^** |  |  |  |  |  |  |
| **Model 1** | | | | | | |
| For pedestrian traffic | 1.00 (0.99-1.00) | 0.54 | 1.00 (0.99-1.01) | 0.69 | 1.00 (0.99-1.00) | 0.64 |
| **Model 2** | | | | | | |
| Sidewalks | 1.00 (0.99-1.00) | 0.72 | 0.98 (0.93-1.03) | 0.39 | 1.00 (0.99-1.00) | 0.97 |
| **Model 3** | | | | | | |
| Free access to people | 1.00 (0.99-1.00) | 0.66 | 0.99 (0.97-1.01) | 0.42 | 1.00 (0.99-1.00) | 0.89 |
| **Model 4** | | | | | | |
| Restricted to vehicles | 1.00 (0.98-1.01) | 0.14 | 0.63 (0.03-12.45) | 0.76 | 0.99 (0.98-1.00) | 0.13 |
| **Model 5** | | | | | | |
| With public lighting | 1.00 (0.99-1.00) | 0.89 | 0.99 (0.96-1.02) | 0.49 | 1.00 (0.99-1.00) | 0.45 |
| **Model 6** | | | | | | |
| Covered with concrete | 1.00 (0.99-1.00) | 0.86 | 0.98 (0.94-1.03) | 0.44 | 1.00 (0.99-1.00) | 0.83 |
| **Model 7** | | | | | | |
| With trees | 1.00 (0.99-1.00) | 0.76 | 0.95 (0.67-1.34) | 0.77 | 1.00 (0.99-1.00) | 0.36 |
| **Model 8** | | | | | | |
| Without peddlers | 1.00 (0.99-1.00) | 0.70 | 1.00 (0.97-1.01) | 0.42 | 1.00 (0.99-1.00) | 0.81 |
| **Neighborhood social environment ^b^** |  |  |  |  |  |  |
| **Model 9** | | | | | | |
| Social capital (score) |  |  |  |  |  |  |
| *Low (0)* | Ref. | | Ref. | | Ref. | |
| *Medium (1)* | 1.21 (0.75-1.97) | 0.44 | 0.98 (0.27-3.56) | 0.97 | 1.09 (0.58-2.06) | 0.80 |
| *High (2-4)* | 1.07 (0.49-2.36) | 0.86 | 2.02 (0.34-11.84) | 0.44 | 0.89 (0.37-2.16) | 0.80 |
| **Model 10** | | | | | | |
| Trust and solidarity |  |  |  |  |  |  |
| *No* | Ref. | | Ref. | | Ref. | |
| *Yes* | 0.94 (0.66-1.34) | 0.72 | 1.24 (0.54-2.84) | 0.61 | 0.78 (0.54-1.14) | 0.20 |
| **Model 11** | | | | | | |
| Safety (score) |  |  |  |  |  |  |
| *High (2-4)* | Ref. | | Ref. | | Ref. | |
| *Medium (1)* | 1.32 (0.95-1.83) | 0.10 | 2.56 (1.08-6.06) | **0.03** | 1.25 (0.82-1.92) | 0.29 |
| *Low (0)* | 1.75 (0.81-3.78) | 0.16 | 1.53 (0.26-8.85) | 0.64 | 1.69 (0.68-4.18) | 0.26 |

^a^ Models with state as the second aggregation level and adjusted for sex, age, depression in wave 1 (baseline), income index, functional limitations and margination index of the municipality. Overall models also adjusted for area of residence.

^b^ Models with state as the second aggregation level and adjusted for age group, sex, depression in wave 1 (baseline), marital status, education level, income quintile, work status, area of residence, social networks, multimorbidity, functional limitations and margination index of the municipality.
