# Supplementary material for: Neighborhood features and depression in Mexican older adults: A longitudinal analysis based on the study on global AGEing and adult health (SAGE), waves 1 and 2 (2009-2014)
Source: PLoS One. 2019 Jul 10;14(7):e0219540. doi: 10.1371/journal.pone.0219540 (PMC6619793; doi:10.1371/journal.pone.0219540)
Supplement: S7 Table — (DOCX) [file pone.0219540.s013.docx]

**S7 Table. Sensitivity analysis of individuals with and without complete information**

| **Variables** | **Complete information** | |  |
| --- | --- | --- | --- |
|  | **No**  **n (%)** | **Yes**  **n (%)** | **p^a^** |
| **Neighborhood physical environment** |  |  |  |
| Total length in meters of space per 100 meters (mean and sd)^b^  n= 326 |  |  |  |
| *For pedestrian traffic* | 441.31 (222.53) | 408.00 (218.75) | **0.02** |
| *Sidewalks* | 276.92 (245.41) | 252.23 (234.62) | 0.11 |
| *Free access to people* | 368.06 (260.00) | 332.74 (250.86) | **0.02** |
| *Restricted to vehicles* | 13.57 (23.48) | 15.04 (25.28) | 0.86 |
| *With public lighting* | 333.96 (244.14) | 306.07 (240.07) | 0.06 |
| *Covered with concrete* | 305.70 (247.30) | 286.40 (242.80) | 0.22 |
| *With trees* | 252.67 (209.92) | 236.67 (206.49) | 0.22 |
| *Without peddlers* | 347.21 (244.87) | 309.99 (233.62) | **0.01** |
| **Neighborhood social environment** |  |  |  |
| Social capital  n= 1260 |  |  | 0.06 |
| *Low* | 197 (74.62) | 802 (80.52) |  |
| *Medium* | 36 (13.64) | 119 (11.95) |  |
| *High* | 31 (11.74) | 75 (7.53) |  |
| Trust and solidarity  n= 1260 |  |  | 0.54 |
| *Yes* | 122 (46.21) | 439 (44.08) |  |
| Safety  n= 1260 |  |  | **0.05** |
| *Low* | 4 (1.52) | 47 (4.72) |  |
| *Medium* | 94 (35.61) | 366 (36.75) |  |
| *High* | 166 (62.88) | 583 (58.53) |  |
| Sex  n= 1301 |  |  | 0.57 |
| *Male* | 126 (41.31) | 430 (43.17) |  |
| Age (years)  n= 1304 |  |  | **<0.001** |
| *55-59* | 47 (15.26) | 181 (18.17) |  |
| *60-69* | 114 (37.01) | 461 (46.29) |  |
| *70-79* | 82 (26.62) | 284 (28.51) |  |
| *80+* | 65 (21.10) | 70 (7.03) |  |
| Marital status  n= 1263 |  |  | 0.10 |
| *Has permanent partner* | 161 (60.30) | 654 (65.66) |  |
| Educational level  n= 1263 |  |  | **<0.001** |
| *No schooling* | 96 (35.96) | 165 (16.57) |  |
| *Complete elementary* | 149 (55.81) | 657 (65.96) |  |
| *Complete high school* | 10 (3.75) | 92 (9.24) |  |
| *Complete college* | 12 (4.49) | 82 (8.23) |  |
| Currently working  n= 1260 |  |  | 0.06 |
| *No* | 69 (26.14) | 269 (27.01) |  |
| *Yes* | 53 (23.86) | 303 (30.42) |  |
| *Has never worked* | 132 (50.00) | 424 (42.57) |  |
| Area of residence  n= 1524 |  |  | **<0.001** |
| *Urban area* | 327 (61.93) | 729 (73.19) |  |
| Multimorbidity  n= 1263 |  |  | 0.65 |
| *Yes* | 81 (30.34) | 288 (28.92) |  |
| Functional limitations  n= 1263 |  |  | **0.02** |
| *Yes* | 125 (46.82) | 386 (38.76) |  |
| SES (quintiles)  n= 1523 |  |  | **0.01** |
| *1* | 143 (27.13) | 198 (19.88) |  |
| *2* | 95 (18.03) | 204 (20.48) |  |
| *3* | 106 (20.11) | 180 (18.07) |  |
| *4* | 97 (18.41) | 221 (22.19) |  |
| *5* | 86 (16.32) | 193 (19.38) |  |
| Social networks  n= 1261 |  |  | 0.33 |
| *0* | 25 (9.43) | 72 (7.23) |  |
| *1* | 86 (32.45) | 331 (33.23) |  |
| *2* | 100 (37.74) | 425 (42.67) |  |
| *3* | 47 (17.74) | 150 (16.06) |  |
| *4* | 7 (2.64) | 18 (1.81) |  |

Results are presented by columns

People with complete information were 996, but since the number of observations (n) is not the same for all variables, it is presented within each variable

^a^ p-values estimated with the χ^2^ and Fisher exact tests for categorical variables and with the Mann-Whitney U test for numeric variables.

^b^ Measurements of the street network buffer. Estimations are presented as total meters divided by a 100 meters

Significance level= 0.05
