# Supplementary material for: Neighborhood features and depression in Mexican older adults: A longitudinal analysis based on the study on global AGEing and adult health (SAGE), waves 1 and 2 (2009-2014)
Source: PLoS One. 2019 Jul 10;14(7):e0219540. doi: 10.1371/journal.pone.0219540 (PMC6619793; doi:10.1371/journal.pone.0219540)
Supplement: S8 Table — (DOCX) [file pone.0219540.s014.docx]

**S8 Table. Sensitivity analysis of logistic regression models accounting for the complex sampling design of SAGE**

| **Urban area (n=729)** | | |
| --- | --- | --- |
| **Neighborhood measurement** | **OR (CI 95%)** | **p** |
| Space with sidewalks (per 100 meters) |  |  |
| *0 to less than 50* | Ref. | |
| *50 to less than 250* | 0.33 (0.10-1.09) | 0.07 |
| *250 to less than 450* | 0.57 (0.17-1.93) | 0.37 |
| *450 or more* | 2.65 (0.82-8.56) | 0.10 |
| Space with tress (per 100 meters) |  |  |
| *0 to less than 100* | Ref. | |
| *100 to less than 300* | 0.45 (0.13-1.55) | 0.20 |
| *300 to less than 500* | 1.65 (0.49-5.56) | 0.41 |
| *500 or more* | 2.05 (0.61-6.96) | 0.25 |
| Space restricted to vehicles (per 100 meters) |  |  |
| *0 to less than 15* | Ref. | |
| *15 to less than 45* | 0.30 (0.10-0.94) | **0.04** |
| *45 to less than 80* | 0.29 (0.08-1.09) | 0.07 |
| *80 or more* | 0.06 (0.01-0.43) | **<0.01** |

Multiple logistic regression models accounting for the complex design of the original study (specification of strata and sample weights) and adjusted for sex, age, income index, functional limitations and deprivation index of the municipality.

Multilevel analysis could not be performed at the same time because the group variable for that analysis did not match
